# Molecular and long-term behavioral consequences of neonatal opioid exposure and withdrawal in mice

**DOI:** 10.3389/fnbeh.2023.1202099

**Published:** 2023-06-21

**Authors:** Amelia D. Dunn, Shivon A. Robinson, Chiso Nwokafor, Molly Estill, Julia Ferrante, Li Shen, Crystal O. Lemchi, Jordi Creus-Muncunill, Angie Ramirez, Juliet Mengaziol, Julia K. Brynildsen, Mark Leggas, Jamie Horn, Michelle E. Ehrlich, Julie A. Blendy

**Affiliations:** ^1^Department of Systems Pharmacology and Translational Therapeutics, Perelman School of Medicine, University of Pennsylvania, Philadelphia, PA, United States; ^2^Department of Psychology, Williams College, Williamstown, MA, United States; ^3^Department of Neurology, Icahn School of Medicine at Mount Sinai, New York, NY, United States; ^4^Department of Neuroscience, Icahn School of Medicine at Mount Sinai, New York, NY, United States; ^5^Department of Bioengineering, School of Engineering and Applied Science, University of Pennsylvania, Philadelphia, PA, United States; ^6^Department of Pharmaceutical Sciences, St. Jude Children’s Research Hospital, Memphis, TN, United States

**Keywords:** opioid, neonatal, withdrawal, brain transcriptome, mouse, behavior

## Abstract

**Introduction:**

Infants exposed to opioids *in utero* are at high risk of exhibiting Neonatal Opioid Withdrawal Syndrome (NOWS), a combination of somatic withdrawal symptoms including high pitched crying, sleeplessness, irritability, gastrointestinal distress, and in the worst cases, seizures. The heterogeneity of *in utero* opioid exposure, particularly exposure to polypharmacy, makes it difficult to investigate the underlying molecular mechanisms that could inform early diagnosis and treatment of NOWS, and challenging to investigate consequences later in life.

**Methods:**

To address these issues, we developed a mouse model of NOWS that includes gestational and post-natal morphine exposure that encompasses the developmental equivalent of all three human trimesters and assessed both behavior and transcriptome alterations.

**Results:**

Opioid exposure throughout all three human equivalent trimesters delayed developmental milestones and produced acute withdrawal phenotypes in mice reminiscent of those observed in infants. We also uncovered different patterns of gene expression depending on the duration and timing of opioid exposure (3-trimesters, *in utero* only, or the last trimester equivalent only). Opioid exposure and subsequent withdrawal affected social behavior and sleep in adulthood in a sex-dependent manner but did not affect adult behaviors related to anxiety, depression, or opioid response.

**Discussion:**

Despite marked withdrawal and delays in development, long-term deficits in behaviors typically associated with substance use disorders were modest. Remarkably, transcriptomic analysis revealed an enrichment for genes with altered expression in published datasets for Autism Spectrum Disorders, which correlate well with the deficits in social affiliation seen in our model. The number of differentially expressed genes between the NOWS and saline groups varied markedly based on exposure protocol and sex, but common pathways included synapse development, the GABAergic and myelin systems, and mitochondrial function.

## Introduction

Opioid use has increased dramatically among all demographics in the US over the past few decades, including in pregnant people. A recent study found that opioid use disorder (OUD) during pregnancy has increased in every state from 2010 to 2017, with levels as high as 40 per 1,000 deliveries in several states (Hirai et al., 2021). Infants exposed to opioids prenatally can develop neonatal abstinence syndrome, now referred to as neonatal opioid withdrawal syndrome (NOWS) after birth. NOWS is a combination of physical withdrawal symptoms including high pitched crying, sleeplessness, irritability, gastrointestinal distress, and in the worst cases, seizures (Logan et al., 2013). NOWS cases almost doubled from 0.4% to 0.73% of birth hospitalizations nationwide from 2010 to 2017 (Hirai et al., 2021). With the increasing incidence of prenatal opioid exposure and NOWS, it is critical to investigate associated molecular underpinnings which may contribute to withdrawal and/or any neurological or behavioral consequences that may persist into adulthood.

Long-term follow-up of infants exposed to opioids *in utero* is difficult because of limited time in treatment and frequent adverse psychosocial experiences later in life. Some clinical studies report that prenatally opioid-exposed children have increased behavioral problems and cognitive impairments compared to those without prenatal exposure (Wilson et al., 1979; Konijnenberg and Melinder, 2015; Nygaard et al., 2016; Benninger et al., 2023). However, clinical studies are unable to clearly dissociate the influence of maternal polypharmacy, parental rearing, or the household environment from direct effects of *in utero* drug exposure (Conradt et al., 2019). For these reasons, animal models are critical tools for investigating the immediate and long-term consequences of opioid exposure and withdrawal. Preclinical rodent models have been developed to study the consequences of *in utero* opioid exposure and, similar to human studies, the results are varied. Prenatal morphine exposure has been shown to impair neuronal development (Ricalde and Hammer, 1990; Mei et al., 2009); as have opioids such as buprenorphine (Wu et al., 2014) and methadone, which also delayed key developmental milestones and induced hyperactivity in young mice (Grecco et al., 2021). Changes in opioid reward behavior in adulthood modeled by either intravenous self-administration (Glick et al., 1977; Ramsey et al., 1993; Riley and Vathy, 2006) or conditioned-place preference (Gagin et al., 1996, 1997) as well as learning and memory impairments (Lin et al., 2009; Davis et al., 2010; Tan et al., 2015) including sex-dependent executive function deficits (Smith et al., 2022) have also been documented following neonatal opioid exposure.

Reasons for divergent outcomes of these preclinical models in both mice and rats likely include variation in the type of opioid employed, the route of administration as well as the duration of opioid exposure which can encompass a portion of gestation (Castellano and Ammassari-Teule, 1984; Gagin et al., 1997; Slamberova et al., 2001; Riley and Vathy, 2006; Tan et al., 2015), all of gestation (Niesink et al., 1999; Bhat et al., 2006; Buisman-Pijlman et al., 2009; Wu et al., 2009), gestation plus a variable postnatal period (Grecco et al., 2021; Smith et al., 2022), or post-natal (PND) treatment only (Cuomo et al., 1988; Barr and Wang, 1992; Boasen et al., 2009; Robinson et al., 2020; Borrelli et al., 2021). Importantly, the central nervous system of a newborn rodent is developmentally analogous to a 24-week (2*^nd^* trimester) human fetus, while the first two post-natal weeks in rodents are analogous to the 3*^rd^* trimester in humans (Dobbing and Sands, 1979; Barr et al., 2011; Semple et al., 2013). In humans, synaptic proliferation begins during the second trimester (Huttenlocher, 1979; Herschkowitz et al., 1999), whereas the critical period of synaptogenesis occurs during the first 3 post-natal weeks in rodents (Semple et al., 2013). Thus, opioid exposure during mouse gestation alone or postnatal alone does not model the full developmental insult experienced by a human fetus exposed to opioids during the entire time *in utero*. Therefore, to determine the impact of opioid exposure throughout the human equivalent of pregnancy, we employed a “3-trimester” equivalent exposure model that delivers consistent doses of opioids to pups throughout development. We found that the combination of gestational and postnatal opioid exposure leads to developmental delays and robust, spontaneous opioid withdrawal after cessation of opioid exposure, recapitulating key features of human NOWS. To better understand the impact of different exposure durations and how these may influence behavior phenotypes, we generated transcriptome signatures from forebrains of males and females. Because changes in gene expression may not persist throughout all three trimesters, we captured differentially expressed genes (DEGs) following *in utero*, third trimester only or all 3-trimester drug exposure. We rationalized that gene expression changes restricted to any of these three periods could impact brain circuitry and thereby developmental delays, withdrawal, and long-term behavior. Overall, the results from these datasets were surprising, as significantly more DEGS were found following *in utero* only exposure, compared to other exposure windows. In addition, with the exception of sex-specific changes in social behavior and sleep behavior in adulthood, we found no deficits in reward or affective behaviors.

## Materials and methods

### Animals

Male and female C57Bl/6NTac mice (referred to as B6 throughout; Taconic, Hudson, New York) were bred at the University of Pennsylvania. Upon weaning, mice were group-housed (3–5/cage) with *ad libitum* access to food and water under a 12-h light/dark cycle (lights on at 0700). All procedures were approved by the University of Pennsylvania Animal Care and Use Committee and were in compliance with the National Institutes of Health Guide for the Care and Use of Laboratory Animals.

### Drugs

Morphine sulfate and remifentanil were obtained from NIDA Drug Supply (Research Triangle Park, NC) and dissolved in 0.9% saline.

### Three trimester morphine exposure paradigm

Osmotic minipumps (model 1004; Alzet, Cupertino CA) providing either 10 mg/kg/day morphine or saline were implanted subcutaneously in 8–13-week-old female mice which were then allowed to recover for 3 days. Implanted females were co-housed with drug naïve B6 male counterparts for 2 weeks for mating. Cages were examined for pups 3x daily. Litters from implanted females were transferred to a surrogate dam within 4–8 h after birth (mice typically give birth between 1:00 am and 4:00 am and first checks were at 8:00 am). Surrogate dams were actively nurturing naïve pups of the same age (+2–4 days). Drug or saline-exposed pups were co-housed with 2 naïve pups for 8–16 h to facilitate surrogate nurturing, after which naïve pups were removed from cage. Third trimester equivalent exposure began on the evening of postnatal day 1 (PND1) and continued until the morning of PND14. Morphine was delivered at a dose of 10 mg/kg in a volume of 10 mL/kg and administered subcutaneously twice daily at 9:00 AM and 5:00 PM. The last injection of morphine was given on the morning of PND14. Developmental milestones were always measured just prior to the 5:00 PM injection. Controls received saline in an equal injection volume. The treatment of the dam determined the pup injection treatment (morphine or saline) during PND 1–14. Four different cohorts of pregnant dams were used to generate the data for this study. Cohort size, and developmental milestones and withdrawal statistics for each cohort are shown in Supplementary Figure 1. From PND 1–21, pup weight was examined daily and developmental milestones including eye opening, surface righting, forelimb grasping, and extinguishing pivoting behavior were assessed as described by Robinson et al. (2020).

### Morphine measurements

After collection, brains were frozen in isopentane and stored at −80 degrees. Morphine levels were quantitated in fetal and pup mouse brain by LC-MSMS detection of the m/z 286.15 to m/z 165.03, which refers to morphine molecular ion to fragment ion transition, in reconstituted protein precipitation extracts from calibrator, quality control and experimental sample 1:1 brain:PBS homogenates. Briefly, excised brain tissue was homogenized and mixed with 0.4x volume equivalent (eq.) of aqueous ISTD solution (714 ng/mL morphine-D3 deuterated standard; Cerilliant Corp., Round Rock, Tx). Protein was precipitated with 14x volume eq. of 50:50 methanol:acetonitrile containing 0.1% formic acid. Samples were prepared for LC-MSMS analysis *via* centrifuge, vacuum drying, and 1x volume eq. reconstitution in water. LC-MSMS analysis was conducted on a Sciex Triple Quad™ 7,500 equipped with a Sciex ExionLC™ AD liquid chromatography system and controlled using Sciex OS software (Ver.2.0). Seven microliters of reconstituted extract were injected onto an Inertsil^®^ ODS-3 (3 × 100 mm; 4 μ) analytical column. The column was maintained at 40°C and was eluted with a gradient of 5% methanol in water containing 0.1% formic acid (Mobile Phase-A) and methanol containing 0.1% formic acid (Mobile Phase-B) at a flow of 0.2 mL/min. The gradient progressed from 0 to 15% MP-B over 2 min then increased to 35% MP-B over 0.5 min. MP-B was maintained at 35% for 3.5 min before being decreased to 0% over 0.5 min. The column was equilibrated at 0% MP-B for 7 min, resulting in a 13.5 min total run time. The eluent was volatilized within an OptiFlow^®^ Pro ion source (fixed position electrode at 2,200 V, temperature 400°C, N2 heater gas at 50 psi, nebulization N2 gas at 30 psi and N2 curtain gas at 40 psi) prior to MRM monitoring for molecular to fragment ion transitions of morphine (m/z 286.2à165.0; EP = 11/CE = 55/CXP = 24; Avg. Rt = 4.29 min). Calibration curves were generated within the OS software by 1/x^2 weighted, linear regression of nominal analyte to ISTD concentration ratios to their respective analyte/ISTD area ratios (y = 0.00127x + 2.89e-4, R2 = 0.998). Morphine concentration data was analyzed with GraphPad Prism 9 (GraphPad Software) and is displayed as mean. Morphine concentration data was analyzed by one-way ANOVA with *post hoc* testing conducted using Bonferroni’s multiple-comparisons correction (Table 1).

**TABLE 1:** Brain morphine measurements.

Collection date	Morphine conc. (ng/g tissue)
ED10 (*n* = 3)	60.77 ± 6.24
ED16 (*n* = 6)	37.19 ± 6.71
ED20 (*n* = 11)	37.32 ± 6.10
PND1 (*n* = 3)	37.67 ± 13.90
PND6 (*n* = 1)	46.35 ± 0.00
Control (*n* = 3)	*Below limit of quantitation*

### Withdrawal behaviors

**FIGURE 1:**
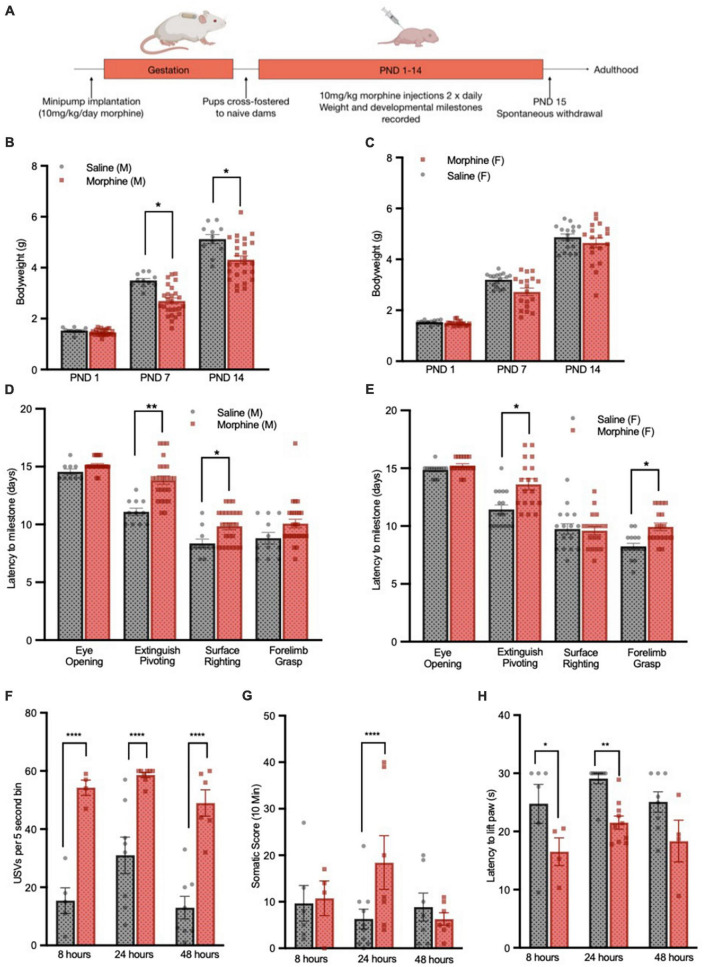
3-Trimester NOWS model recapitulates delayed development and weight gain deficits. **(A)** Schematic of 3-trimester NOWS model. Weights of male **(B)** and female **(C)** pups through the first 14 days of life. For male mice, there was a modest main effect of NOWS treatment [F(1,9.8) = 4.5, *p* < 0.1], and a significant time x treatment interaction [F(2,70) = 9.7, *p* < 0.001]. *Post hoc* testing revealed a significant difference between saline and NOWS groups at PND 7 [χ2(1,N = 37) = 8.24, *p* < 0.05] and PND 14 [χ2(1,N = 37) = 8.03, *p* < 0.05]. For females, there was no main effect of NOWS treatment, but there was a significant time x treatment interaction [F(2,86.4) = 3.3, *p* < 0.05]. Time to reach developmental milestones for male **(D)** and female **(E)** pups during the first 2 weeks of life. For male mice, there was a significant effect of NOWS treatment on the time to extinguish pivoting behavior [F(1,9.6) = 13.8, *p* < 0.01] and time to surface righting [F(1,11.5) = 4.9, *p* < 0.05]. For female mice, there was a significant effect of NOWS treatment on the time to extinguish pivoting behavior [F(1,8.9) = 7.1, *p* < 0.05] and time to forelimb grasping [F(1,6.5) = 10.8, *p* < 0.05]. **(F)** USV emission, measured by the number of 5 s bins containing a USV during the 5-min test period. There was a significant NOWS treatment x time interaction [χ2(2,N = 39) = 32.08, *p* < 0.001]. *Post hoc* testing revealed a significant difference between saline and NOWS groups at 8 h [χ2(1,N = 9) = 47.23, *p* < 0.0001], 24 h [χ2(1,N = 16) = 19.27, *p* < 0.0001] and 48 h [χ2(1,N = 14) = 60.27, *p* < 0.0001]. **(G)**Total score of somatic signs of withdrawal during 10-min test period. There was a significant NOWS treatment x time interaction [χ2(2,N = 40) = 52.10, *p* < 0.001]. *Post hoc* testing revealed a significant difference between saline and NOWS groups at 24 h [χ2(1,N = 16) = 18.87, *p* < 0.0001]. *p < 0.05, **p < 0.01, ****p < 0.0001. All data was generated from 7 total litters (3 saline, 4 NOWS). **(H)** Latency to lift hind paw or jump on a 55*^o^*C hotplate assay, at select timepoints after last injection. There was a main effect of NOWS treatment [F(2,35) = 18.0, *p* < 0.001]. *Post hoc* testing revealed a significant difference between saline and NOWS groups at 8 h [χ2(1,N = 10) = 11.32, *p* < 0.01] and 24 h [χ2(1,N = 19) = 6.14, *p* < 0.05].

Ultrasonic vocalizations, somatic withdrawal, and hot plate latency measurements were taken at 8, 24, and 48 h post the final injection of saline or morphine, with different pups being tested at each timepoint. Individual pups were separated from the dam and littermates and placed in a petri dish with fresh bedding. Ultrasonic vocalizations were recorded for 5 min as described in Robinson et al. (2020). DeepSqueak software was used to perform spectrographic analysis of USVs (Coffey et al., 2019). A novel detection network was made using USVs from a previous cohort of over 50 pups, based on the DeepSqueak Mouse Call Network, to detect USVs from each recording. This network did not detect call type. Next, the pup was transferred to a clean 300 ml glass beaker in a different room. Pup activity was filmed for 10 min and later scored for total numbers of wall-climbs (forepaws moving back and forth against wall of beaker) and jumps (all four paws of the ground). The frequency of each behavior was summed to create the withdrawal score. Finally, nociceptive sensitivity was measured on a 55°C hot plate. Latency to show any of the following behaviors within 30 s was measured: licking hind paws, wall climbing, sudden shaking of hind paws, and jumps. The first behavior observed was considered the latency score. Data shown in Figure 1 are from several different timepoints to determine the optimal time to evaluate withdrawal behaviors. Based on this timecourse, all subsequent withdrawal data (Supplementary Figure 1) are from 24 h after the last morphine injection.

### Adult behaviors

For all adult behaviors, mice were group housed (*N* = 3–5 per cage) and were tested between 12 and 25 weeks of age. Mice were acclimated to testing rooms for at least 30 min prior to the test for all assays except self-administration. For self-administration studies, mice were housed in the same room as Med Associate operant chambers immediately following surgery. Cohort 2 was used for EPM, TST and Learned Helplessness and tested in that order with 2 days between each test. Cohort 3 was split, with half the mice used for CPP and the other half used for self-administration.

#### Elevated plus maze (EPM)

The “ + “ shaped maze has two oppositely positioned closed arms, two oppositely positioned open arms, and a center area and stands 60.0 cm above the floor. Mice were placed facing the closed arm quadrant and allowed unlimited exploration of all 4 quadrants for 5 min under dim lighting (50 lux). Latency to emerge from the closed arm, the amount of time spent in the open arms, and transitions between arms were quantified by a trained blinded observer.

#### Tail suspension test–(TST)

Mice were suspended by their tails with tape and elevated 16 cm above a tabletop (1–2 cm of tail remaining outside of the tape), for 6 min. Individual mice were separated by vertical black plexiglass dividers. The test was video recorded and scored by a trained blinded observer for time spent immobile.

#### Learned helplessness

Mice were trained for 30 min in a single closed chamber (Med Associates, St. Albens, VT, USA) with inescapable foot-shocks of 0.3 mA intensity with random inter-shock intervals. The subsequent day, mice were tested in the same apparatus with an open door between the two chambers. Mice were delivered 25 shock trials during the test, and the time to escape the foot-shock was measured.

#### Conditioned place preference

Mice were tested as previously described (Walters et al., 2005). Briefly, conditioning was performed using an unbiased, counterbalanced design. Animals that spent more than 700 s in either side of the apparatus during the pretest were excluded from the remainder of the study. On conditioning days, mice were injected with morphine (20 mg/kg; morphine sulfate, NIDA Drug Supply, Research Triangle Park, NC) or saline and immediately confined to one side of the apparatus. Preference was then measured 1 day after the last conditioning session. Pretest and Test sessions were 15 min in duration, and conditioning sessions were 30 min in duration. To assess locomotor activity, beam breaks were recorded using MED Associates software during each 30-min saline and morphine conditioning session. Activity counts were summed within each conditioning session and compared across sessions for each animal.

#### Self-administration

Mice underwent jugular catheterization surgery and were allowed to recover for 5 days. Patency was then confirmed *via* a ketamine infusion. Mice were trained in operant chambers (Med Associates, St. Albens, VT, USA) to intravenously self-administer remifentanil (0.1 mg/kg/infusion) through paired cues (tone and light) to active and inactive nose pokes. Following completion of training criteria (at least 50 infusions during a 12-h session and at least 10 infusions in consecutive 2-h training sessions), mice proceeded to a fixed-ratio (FR) 1 schedule of reinforcement for 6 days, then FR2 for 4 days followed by a progressive ratio schedule of reinforcement [m = (5*EXP(x*0.2)]-5, where m is the next FR requirement and x is the injection number to be earned).

#### 3-chamber social affiliation

Mice were acclimated for 10 min to an empty 3-chamber apparatus. Gonadectomized A/J stimulus mice (age-and sex-matched to experimental mice) were placed under a plastic cylinder with holes (social cylinder) and a novel object (purple plastic paper weight) was placed under an identical plastic cylinder (non-social cylinder). The side-placement of social and non-social cylinders were randomized and balanced across test groups. EthoVision XT16 tracking software (RRID:SCR_000441, Noldus, Netherlands) was used to measure time spent interacting with the social and non-social cylinders, where time interacting is defined as the mouse being within 1 cm of the cylinder.

#### Sleep

Mice were removed from their home cage and singly housed in clean cages resembling the home cage (26 × 20 × 14 cm) in a new room at the beginning of the light cycle (between 9 and 10 am). The Continuous Open Mouse Phenotyping of Activity and Sleep Status (COMPASS) system uses passive infrared sensors which are attached to the cage top to monitor and document activity continuously *via* an open-source electronics platform which records active movement by receiving inputs from multiple sensors (Arduino). Activity is collected and the software generates a score from 0 to 100 based on 10 ms recordings for a bin every 10 s, denoting the percent of time mice were moving in that 10 s bin. Extended inactivity (0 score) greater than 40 s correlates with sleep measured by electroencephalography with a Pearson coefficient of greater than 0.95 (Brown et al., 2016). We used a custom program in Python to convert 40 s of inactivity into sleep and to identify bouts of sleep that lasted longer than 40 s. Data analysis began at the first dark cycle with at least 8 h of acclimation time as described in Brown et al. (2016).

### RNA sequencing

Brains were dissected from PND 1 pups 6 h after discovery. Brains were dissected from post-natal exposure only (PND 14) or 3-trimester exposure (3-tri) 6 h after the last morphine or saline injection. This time point was selected to equate morphine levels among all groups at time of death. Total RNA was extracted from the right brain hemisphere using the miRNeasy mini kit (Qiagen, Netherlands) and RNA integrity assessed in an Agilent Tapestation 4200 using the RNA ScreenTape (Agilent, Wilmington, DE). All samples had RIN values > 8. mRNA libraries were generated using the NEBNext Ultra™ II RNA Library Prep Kit for Illumina (New England BioLabs, Ipswitch, MA). The number of animals per group was similar (*N* = 5–7 animals), and the quality controls, library construction and sequence parameters were also identical across all groups.

Libraries were sequenced on a NovaSeq 6000 at a depth of 30 million total reads/sample using paired-end sequencing of 150 base pairs (PE150), to a depth of 30 million total reads/sample. Reads were processed using the NGS-Data-Charmer pipeline.^1^ In brief, the pipeline removed adaptors and low-quality bases from the reads using Trim Galore.^2^ Reads were then mapped to the mouse reference genome (Mus Musculus, GRCm38/mm10) using HISAT2 (version 2.2.1), and duplicated fragments were removed using Picard MarkDuplicates.^3^ Read counts for the GENCODE gene annotation (version M25) (Frankish et al., 2023) were generated using featureCounts (v2.0.1).

Differential expression analysis between two conditions (e.g., Morphine and Saline) was performed in R (version 4.1.1) with DESeq2 (v1.32.0) package. In brief, DESeq2 provides statistical platform for determining differential gene expression using a model based on the negative binomial distribution. Genes were assigned as differentially expressed if the (adjusted) (nominal) *p*-value < 0.05. Gene ontology (GO) analyses were performed *via* ClusterProfiler (v3.8.1). The Canonical Pathways and Upstream regulators were generated through the use of Ingenuity Pathway Analysis (IPA; QIAGEN Inc.^4^). Enrichment for Autism Spectrum Disorder (ASD) gene sets was assessed using Fisher’s exact tests, and *P*-values were adjusted using the Benjamini-Hochberg method.

### Real-time qPCR

For gene expression, snap-frozen samples were homogenized in QIAzol Lysis Reagent (Qiagen). Total RNA was extracted from one brain hemisphere with TriZol reagent (Invitrogen) and purified with the miRNeasy mini kit according to the manufacturer’s instructions (Qiagen), nano drop purity was A260/280 = 1.8–2.2 and A260/230 ≥ 1.8. 1000 ng of each RNA were reverse-transcribed using the High Capacity RNA-to-cDNA Kit (Applied Biosystems, Foster City, CA, USA). Real-time qPCR was performed in a Step-One Plus system (Applied Biosystems) using TaqMan assays following manufacturer protocol. The following Taqman probes were used in this study: GAPDH (Mm99999915_g1), Gad2 (Mm00484623_m1), Mag (Mm00487538_m1), Myrf (Mm01194959_m1). The individual value for each gene, performed in duplicate, was normalized to GAPDH levels. Relative quantification was performed using the ΔΔCt method (Livak and Schmittgen, 2001) and was expressed as fold change relative to control by calculating 2-ΔΔCt.

### Statistics

Behavioral data were analyzed using mixed effects models. Non-normal count data (EPM entries into open arms and failures to escape in the learned helplessness paradigm) were analyzed using generalized linear mixed effects models with a Poisson probability distribution and a log link. All other data were analyzed using linear mixed effects models. Models for developmental milestones, time in open arms of the EPM, time immobile in the TST, PR breakpoint, and nest shredding included fixed effects of drug treatment (morphine or saline). Models for body weight, withdrawal behaviors, learned helplessness, CPP, IVSA over trials, activity during morphine conditioning sessions, social affiliation, and sleep data included fixed effects of treatment and timepoint (day, hour, trial, or session) and their interaction. All models included litter as a random effect and repeated measures designs also included subject as a random effect. Mixed effects modeling was performed within R (R Core Team, 2021) using package “lme4” (Bates et al., 2015), *p* values were obtained using “afex” (Singmann et al., 2021) and “lmerTest” (Haubo, 2021), and *post hoc* tests were performed using package “phia” (De Rosario-Martinez, 2015) with the Holm-Bonferroni correction for multiple comparisons. A *p* < 0.05 was considered statistically significant. Psuedo-R^2^ values were calculated from the mixed effects models (Nakagawa and Schielzeth, 2013). Effect sizes are reported as the marginal R^2^m value, which takes into account the model fixed effects only.

## Results

### Brain morphine levels

A separate cohort of mice was used to quantify morphine concentrations in pups throughout gestation and in the early post-natal period. Mice underwent the 3-trimester exposure paradigm as described in Section “Materials and methods” and pup brain tissue was collected at the following timepoints: embryonic day (ED) 10, ED16, ED20, post-natal day (PND) 1, and PND6. For all embryonic timepoints, pups and brain tissue were collected *via* cesarean section at 9 AM of that day. For ED10 6 embryos were collected, and two brains pooled to produce a total of 3 samples for analysis. For ED16, 12 embryos were collected from two dams and 2 brains were pooled to produce a total of 6 samples. By ED 20, brains were collected from 3 dams and were of sufficient weight to use as individual samples. For post-natal timepoints, brains were collected across two different litters, 6 h after their final morphine injection. Control brains were collected from PND14 mice who did not have any morphine exposure. No significant differences were found between experimental groups, and all experimental groups had significantly higher morphine concentrations compared to controls (*p* < 0.05). The higher morphine levels observed in ED10 may reflect a decreasing amount of morphine being delivered as the dam gains weight over pregnancy.

### “3-trimester” opioid exposure leads to developmental delays and a robust opioid withdrawal response

Following 3-trimester opioid or saline exposure, (Figure 1A), male but not female morphine-treated pups had significantly lower body weights (Figures 1B, C) which persisted through PND 21 but normalized once mice reached adulthood at 12 weeks (data not shown). In subsequent cohorts both sexes showed significant weight loss (Supplementary Figure 1). Additionally, morphine-treated pups of both sexes took longer to reach several developmental milestones, including surface righting, grasping a rod with forelimbs, and extinguishing of pivoting behavior (Figures 1D, E).

After the last morphine injection on the morning of PND14, there were no sex differences in withdrawal behaviors (data not shown), and therefore mixed-sex groups were used to assess signs of spontaneous withdrawal at 8 h, 24 h, and 48 h. Morphine-treated mice had increased ultrasonic vocalizations (USVs) when separated from the litter, beginning at 8 h after the last injection (Figure 1F), but the full withdrawal syndrome did not emerge until 24 h after the last injection. Morphine-treated mice showed increased somatic signs of withdrawal (Figure 1G) and increased thermal sensitivity, measured by decreased latency to remove a paw or jump in a hotplate assay (Figure 1H). As all withdrawal signs were significantly different at the 24 h time point, subsequent cohorts of mice used for adult behavior studies were tested for withdrawal at this time and showed similar developmental delays and withdrawal behavior (Supplementary Figure 1; characteristics of all cohorts used in this study).

The robust withdrawal behavior seen after opioid exposure in our paradigm mimics key features of human neonatal opioid withdrawal syndrome (NOWS). For behavioral studies, mice who were exposed to 3-trimesters of morphine and experienced subsequent withdrawal will be referred to as “NOWS mice,” and their saline-exposed counterparts as “saline mice.” For transcriptome studies, mice treated only *in utero* will be described as “PND1” and those treated only post-natally from PND 1–14 as “PND14” and mice treated for all 3-trimseters but not assayed for withdrawal behaviors as “3-Tri.”

### Comparison of transcriptomes across all exposure windows

To examine forebrain transcriptomic changes that might elucidate mechanisms of withdrawal, delayed development, and any long-term behavior changes, we generated transcriptomic signatures following our “3-trimester” exposure model (3-Tri). In addition, we also examined transcriptomes from animals that received opioids only during the gestational period (PND1) or only during the last trimester from PND 1–14 (PND 14). We sought to determine whether transcriptomic signatures vary based on the window of exposure, perhaps contributing to the discrepancies in the literature regarding acute and long-term outcomes. We have previously reported on the withdrawal and adult behavior profile of PND14 mice (Robinson et al., 2020).

**FIGURE 2:**
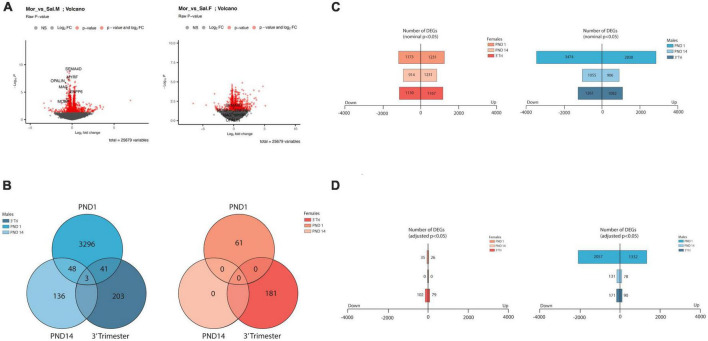
Overview of transcriptome analysis **(A)** Volcano plots of DEGS from RNA seq from male and female PND14 mice. Log2 fold changes are in the same value range observed for all 6 datasets. In these plots, significant and non-significant myelin-associated genes are highlighted, discussed in more detail in the text. **(B)** Venn diagrams showing low numbers of overlapping DEGs (Adj *p* value < 0.05) across treatment group (labeled) and across sex. **(C,D)** Bar graphs showing total number of DEGS in males and females from all 3 exposure protocols. Top graph numbers represent DEGs with nominal significance *p* < 0.05 whereas bottom graphs represent DEGs with adjusted *p* < 0.05 **(D)**.

As evidenced in representative Volcano plots (Figure 2A), the majority of the genes exhibited a low amplitude of change in expression level, with most between log2FC (+)0.50 to (-(-)0.50 in all exposure paradigms. Following all three opioid exposure protocols there were a greater number of differentially expressed genes (DEGs) in RNA derived from males than from females (Figures 2B–D), with no DEGs meeting criteria for adjPvalue < 0.05 in the PND14 females (Figure 2D). Therefore, in subsequent analyses nominal *P* values were sometimes used, and the cutoff is presented for each analysis. Cutoffs for log2FC were not utilized due to the small amplitude of transcript abundance changes. We also noted a greater number of DEGs following exposure restricted to the *in utero* period (PND1) relative to post-natal only (PND14) or 3-trimester (3-Tri). These data imply that gene expression partially normalized post-natally even in the presence of continued morphine exposure. Although these data do not allow for a definitive mechanism for this phenomenon, it is possible that the presence of early life stressors associated with opioid exposure protocols contributes to the alteration in transcriptional programs. Not only did gene expression largely normalize with continued exposure after birth, but the specific DEGs markedly changed (Figure 2B and Supplementary DEG lists). Using the pAdj cutoff of < 0.05, comparison between males in all three exposure protocols showed only 3 DEGs common to all. These 3 genes, *Cobl*, *Parm1*, and *Ust*, play critical roles in neuronal development, including regulation of the axonal growth cone, cortical actin cytoskeleton, axon dendrite development and embryonic axis specification. There were no overlapping DEGs amongst the female groups. As there were no differentially expressed genes in the female PND14 group using the pAdj cutoff of < 0.05, we compared DEGs between males and females in either PND1 or 3-Tri exposure. There were a number of shared DEGs in both PND1 and in the 3-trimester cohort, the comparison with the highest level of overlap between groups when measured by percentage of total DEGs (Supplementary Figure 2).

**FIGURE 3:**
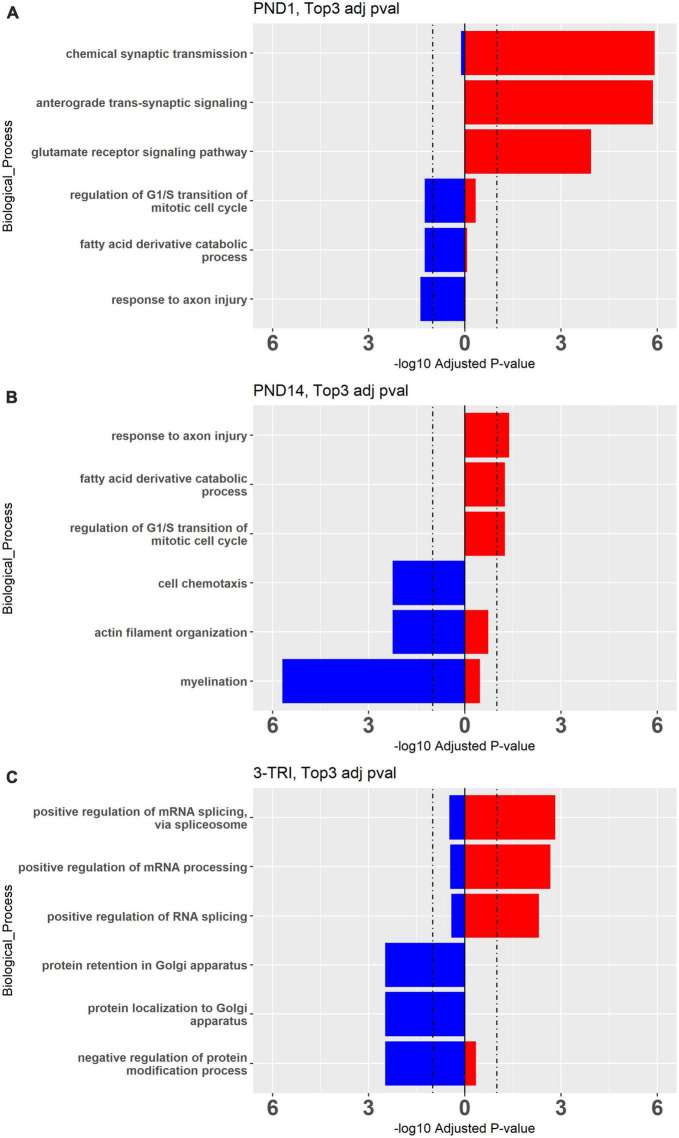
Top 3 Gene Ontology Biological Processes Up- and Down-regulated processes in males from **(A)** PND 1, **(B)** PND14, and **(C)** 3-TRI. Red bars represent upregulated and blue bars represent down regulated genes. Cutoff pAdj < 0.05 for DEGs, showing adjPvalue on X axes, cutoff = 1.2.

**FIGURE 4:**
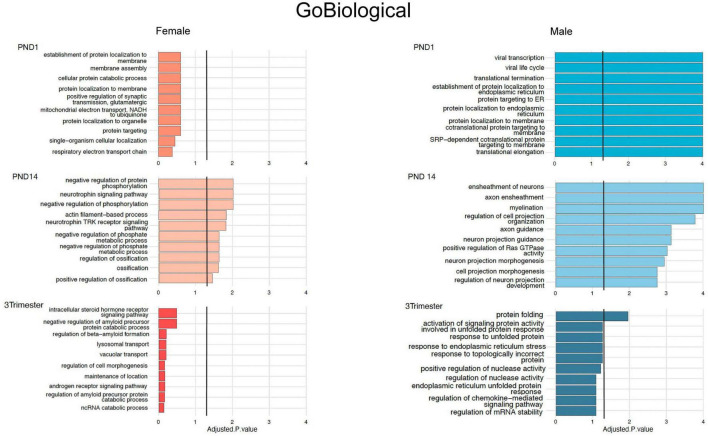
Top 10 Gene Ontology Biological Processes for females (orange bars) and males (blue bars) for all 3 exposure protocols, using nominal *P* values < 0.01, and combining up- and down-regulated DEGs.

To explore the biologic implications of the altered gene expression, we performed Gene Ontology (GO) analysis (Ashburner et al., 2000). First, using the cutoff pAdj < 0.5 as was used for the Venn diagrams, we looked at individual sets of up and downregulated genes, restricted to males in which this analysis was possible in all three exposure paradigms (Figure 3). Notably, there was some overlap between groups for the top 3 up or downregulated, enriched pathways, but in opposite directions, e.g., “response to axon injury” was downregulated in PND1 and upregulated in PND14. To incorporate all three exposure paradigms and both sexes (6 groups) in the GO analysis, we relaxed the stringency and included all DEGs meeting the criteria of a nominal *p* value < 0.01. GO Biological Processes confirmed what was evident from the analysis of individual DEGs, in that most of the enriched pathways in the females do not reach the significance cutoff and overlap between the groups for the top hits was limited as anticipated (Figure 4, females left and males right and Supplementary Figures 4, 5). In the males, in both the PND1 and 3-trimester cohorts, there was an enrichment for genes involved in processing of proteins, including translation, quality control, localization and the role of the endoplasmic reticulum, albeit not in identical GO Biological Processes. There was essentially no overlap with the PND14 male cohort, in which enriched pathways included myelination and neuron projection morphogenesis and guidance. Other recurring themes and terms in the Cellular Component and Molecular Function enriched GO categories included the extracellular matrix, ribosome, mitochondria, and pre- and post-synaptic membrane (Supplementary Figures 4, 5).

**FIGURE 5:**
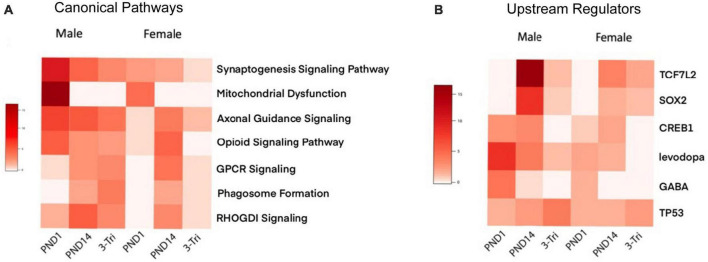
Ingenuity Pathway Analysis **(A)** Comparison of select IPA canonical pathways across exposure protocols and sex. Heatmap of adjusted *P*-values, with rows representing Canonical pathways, and the exposure protocol and sex-segregated models shown as columns. The negative log10 of the adjusted *P*-values is shown in shades of red, with darker reds representing a smaller *p*-value. **(B)** Comparison of select IPA Upstream Regulators across exposure protocols and sex. Heatmap of adjusted *P*-values, with rows representing Canonical pathways, and the exposure protocol and sex-segregated models shown as columns. The negative log10 of the adjusted *P*-values is shown in shades of red, with darker reds representing a smaller *p*-value.

Enrichment of GABA-related processes was restricted to the male PND1 cohort in both the GO Molecular and Cellular categories, i.e., synaptic, while myelin-related processes were enriched in the PND14 male cohort. To further compare and contrast the biologic significance of the DEGs in the three exposure windows, we used Ingenuity Pathway Analysis (IPA), again with relaxed stringency, to identify Canonical Pathways and Upstream Regulators (selected pathways and regulators are shown in Figures 5A, B, and the top pathways and regulators are shown in Supplementary Figures 5A, B). Importantly, the IPA and GO analyses showed significant overlap of pathways. For example, the top Canonical Pathways (Figure 5A and Supplementary Figure 6) identified Opioid Signaling Pathway as significantly enriched in 5/6 groups. RAS GTPase binding and activity, which is required for the effect of opioids on development *via Nf1* (Xie et al., 2016), was prominent in the GO Biological pathways enriched in the PND14 males and in overlapping DEGs between the male groups. Furthermore, Axonal Guidance Signaling was enriched throughout all cohorts in the IPA analysis, as was Protein Kinase A Signaling, critical for opioid and dopamine signal transduction (Svenningsson et al., 2005). The PND1 group remains notable for a high level of enrichment for mitochondrial functions, the activity of which is increasingly linked to neurodevelopment (Mahony and O’Ryan, 2021). Finally, we observed that GABA is a significant Upstream Regulator in the PND1 female and PND14 male cohorts, along with the PND1 males.

To complete the comparisons between groups, we used the Rank Rank Hypergeometric Algorithm (RRHO) which compares gene lists without use of a threshold (Plaisier et al., 2010). There was no concordance noted between any of the female groups (not shown), but there was a moderate degree of discordance between PND1 and 3-trimester, and a high degree of concordance between PND14 and 3-trimester (Supplementary Figure 7), consistent with the IPA Canonical Pathway analysis.

### Enrichment of GABA-related genes and pathways highlights overlap with genes associated with autistic spectrum disorder

**FIGURE 6:**
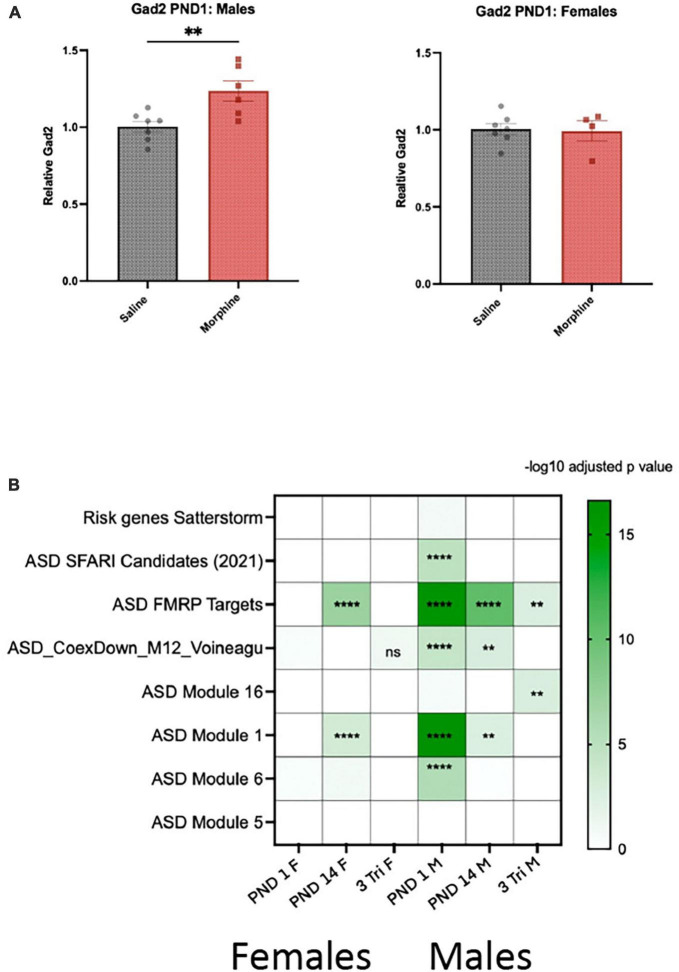
Gene expression and enrichment in ASD datasets **(A)** Gad2 mRNA was assayed by qPCR in PND1 males and females exposed *in utero* to either morphine or saline (*N* = 7M, saline; 6M, morphine; 7Fm saline; 4F, morphine). 2-tail *t*-test, *p* < 0.01 for males, N.S. for females. **(B)** Heatmap showing the FDR for the overlap between DEGs identified in females and males and genes related to autism. Overlap was computed using Fisher’s exact test. Color indicates the -log10 FDR obtained after the correction with Benjamini-Hochberg method. Overlaid asterisks indicate the significance as following: **FDR < 0.01; ****FDR < 0.0001. “ASD Candidates SFARI (2021)”: ASD genetic risk factors from the SFARI database (gene scores 1 to 3 and syndromic); from Abrahams et al.; “Risk genes Satterstorm”: high confidence risk genes described by Satterstrom et al.;“ASD_FMRPTargets”: FMRP (Fragile X mental retardation protein) binding targets from Darnell et al.; “ASD Module 12”: ASD-associated co-expression module 12 and “ASD Module 16”: ASD-associated co-expression module 16 from Voineagu et al.; “ASD Module 1”: ASD-associated co-expression module 1 and “ASD Module 6”: ASD-associated co-expression module 6 and “ASD Module 5”: ASD-associated co-expression module 5 from Gupta et al.

There was a preponderance of enriched GO pathways relevant to GABA in males and/or the synapse in both sexes, which have been implicated in neurodevelopmental abnormalities, particularly autism spectrum disorder (ASD) (Di et al., 2020). qPCR confirmed that *Gad2* mRNA was increased by 25% in PND1 males but not females (Figure 6A). We therefore analyzed the intersection between our gene lists and those that catalog evidence of association with ASD. Using ASD human cortical transcriptomic gene sets comprising differentially expressed genes and ASD-associated co-expression modules, we detected significant enrichments overall in male DEG lists across all exposure windows *via* a Fisher’s Exact Test (Figure 6B). We observed enrichment of male and female DEGs with co-expression module M1 (Gupta et al., 2014) in PND 1 and PND 14. This module is enriched for neuronal markers and synaptic genes and, importantly, is downregulated in post-mortem brains from autistic patients. We also found an enrichment of male DEGs with module M6 from the same study, and ASD genetic risk factors from the SFARI database (Abrahams et al., 2013), but only in PND 1 males; this dataset was also enriched for neuronal markers but upregulated in post-mortem brains from autistic patients. We also observed an enrichment of DEGs from males in the 3-Tri exposure paradigm with module M16 from a different study (Voineagu et al., 2011), which contains genes involved in immune and inflammatory responses and is upregulated in autistic brains. Lastly, we detected a significant enrichment of DEGs in both males and females with targets of FMRP (Darnell et al., 2011), the protein that when mutated causes Fragile-X syndrome. These points to a potential disruption of FMRP function caused by morphine exposure. Only two of the ASD databases showed no enrichment; ASD Module 5 (Gupta et al., 2014) and Satterstrom (Satterstrom et al., 2020).

### Axonal Guidance Signaling pathways are impacted by all 3 morphine exposure paradigms

**FIGURE 7:**
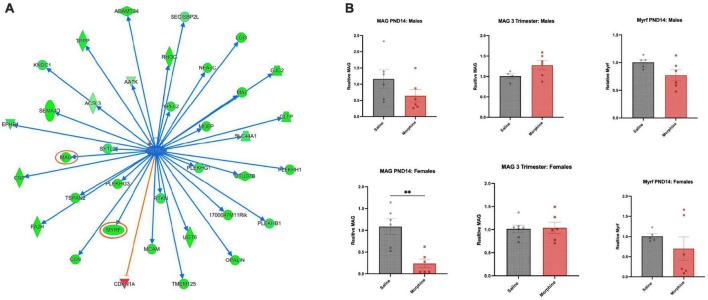
Common regulator and gene validation **(A)** Common upstream regulator Tcf7l2, a transcription factor, is an IPA-predicted upstream regulator across multiple groups and shown here are genes predicted to be regulated in PND14 males (green = down; red = up). The graphs and networks of Tcf7l2 was obtained through the use of IPA (QIAGEN Inc., https://www.qiagenbioinformatics./com/products/ingenuity-pathway-analysis, accessed on 5 February 2023). **(B)** DEG validation *Mag and Myrf* mRNAs were assayed in PND14 males and females after saline or morphine exposure, and *Mag mRNA* was assayed in 3-trimester males and females, saline and morphine exposed. *N* = 4–7; 2-tail *t*-test, ^**^p < 0.01, otherwise not significant.

In the PND14 cohort, one of the most highly enriched GO Biological Process pathways in males was Myelination, and in the IPA Canonical Pathway analysis, Axonal Guidance Signaling was very prominent in multiple cohorts. Analysis of IPA Upstream Regulators highlighted *Tcf7l2* (Figure 7A) and *Sox2*, both known to be involved in myelination and/or oligodendroglial differentiation (Fu et al., 2012; Zhang S. et al., 2018). The top DEGs in both sexes following all three opioid exposures contained multiple, but different, myelin-associated genes, most prominently demonstrated by the IPA map of DEGs regulated by *Tcf7l2* in PND14 males, almost all of which were downregulated, indicated in green. *Mbp*, encoding myelin basic protein, narrowly missed the cutoff, with a log2FC of (-)0.40 and a an adjPvalue = 0.06.

We assayed several of the DEGs using qPCR, with the knowledge that qPCR validation of DEGs derived from bulk RNA preparation and with such low log2FC values is notoriously difficult. Myelin regulatory factor (*Myrf*), a transcription factor required for late stages of oligodendroglial differentiation (Emery et al., 2009), trended toward down-regulation in morphine treated PND14 males. Surprisingly, myelin associated glycoprotein (*Mag*), a constituent of compact myelin which inhibits neurite outgrowth (Quarles, 2007), was unchanged in morphine treated PND14 males, but significantly downregulated in PND14 females, despite the fact that it was unchanged in the RNA sequencing analysis in females. Consistent with the RNA sequencing data, *Myrf* and *Mag* mRNA levels were unchanged in both the male and female 3-trimester cohorts (Figure 7B).

### 3-trimester NOWS does not alter affective behaviors in adulthood

We proceeded to assay behaviors commonly examined in animal models of *in utero*/perinatal opioid exposure, with the knowledge that analysis of the RNA seq data yielded data consistent with an impact on developmental pathways, including neuronal circuitry, that might alter adult behavior. Prior work had reported that gestational exposure to opioids increases anxiety- and depressive-like behavior in adolescence and adulthood (Klausz et al., 2011; Ahmadalipour et al., 2015), while others have reported the opposite or no effect (Walters et al., 2005; McPherson et al., 2007; Tan et al., 2015). Therefore, we investigated behaviors related to these phenotypes in our NOWS mice using the elevated zero maze, forced swim test, and development of learned helplessness.

**FIGURE 8:**
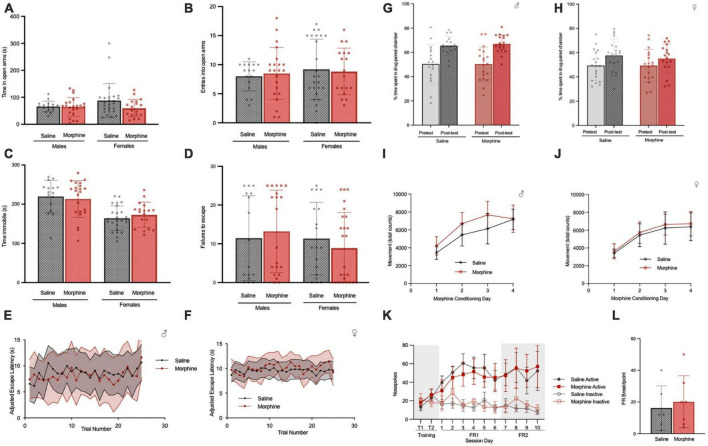
3-trimester NOWS does not alter affective behavior or reward/reinstatement in adulthood. **(A)** Time spent in open arms in the elevated zero, during 5-min total test. For males, there was no effect of NOWS treatment (Effect size = 0.002). For females, there was a modest effect of NOWS treatment [F(1,41) = 2.9, *p* < 0.1]. **(B)** Entries into the open arm during elevated zero maze. There was no effect of NOWS treatment in either sex (Males, Effect Size = 0.008, Females, Effect Size = 0.02). **(C)** Time immobile in the tail-suspension test. There was no effect by NOWS treatment in either sex (Males, Effect Size = 0.005; Females, Effect Size = 0.04). **(D)** Number of failures to escape foot shock in a learned helplessness test. There was no effect of NOWS treatment in either sex (Males, Effect Size = 0.005; Females, Effect Size = 0.02). **(E,F)** Escape latency over 25 trials in the learned helplessness test. There was no effect of NOWS treatment in males (Effect size = 0.03) or females (Effect size = 0.02). **(G)** Morphine conditioned-place preference in male adult mice. There was a significant effect of CPP session [F(1,34) = 36.1, *p* < 0.001] but no effect of NOWS treatment. **(H)** Morphine conditioned-place preference in female adult mice. There was a significant effect of CPP session [F (1,38) = 8.4, *p* < 0.01], but no effect of NOWS treatment. **(I)** Locomotor sensitization during CPP morphine conditioning sessions in male mice. There was a significant effect of conditioning session [F(3,105), *p* < 0.001], but no effect of NOWS treatment. There was a modest NOWS x session interaction [F(3,105) = 2.3, *p* < 0.1]. **(J)** Locomotor sensitization during CPP morphine conditioning sessions in female mice. There was a significant effect of conditioning session [F(3,117) = 145.3, *p* < 0.001], but no effect of NOWS treatment. **(K)** Number of nose pokes in daily 2-h remifentanil (0.1 mg/kg/infusion) IV-self-administration sessions. Sessions included 2 training sessions in which both nose pokes delivered drug infusions, 6 sessions of FR1 reinforcement and 4 sessions of FR2 reinforcement. There was a significant effect of session [F(10,110) = 11.4, *p* < 0.001], but no effect of NOWS treatment. **(L)** Breakpoint for progressive ratio with 0.1 mg/kg/infusion remifentanil. There was no effect of NOWS treatment on PR breakpoint. All data was generated from 16 total litters (6 saline, 10 NOWS).

In the elevated zero maze, there was no difference in the time spent in the open arms between adult saline and NOWS mice of either sex (Figure 8A) or in the number of entries into the open arms (Figure 8B). In the tail suspension test, there was no difference in the time spent immobile between saline and NOWS mice of either sex (Figure 8C). In a learned helplessness paradigm, there was no difference between saline and NOWS mice of either sex in the number of failures to escape (Figure 8D) or the average escape latency across trials in the test period (Figures 8E, F).

### 3-trimester NOWS does not alter response to opioids in adulthood

Saline and NOWS adult mice were tested for morphine reward using the conditioned place preference (CPP) procedure. Using a dose of morphine previously shown to elicit CPP (Walters et al., 2005), we found both saline and NOWS mice exhibit a robust preference for the morphine paired side of the chamber regardless of sex (Figure 8G, H). Additionally, there was no difference in the development of locomotor sensitization during CPP conditioning sessions in either male (Figure 8I) or female mice (Figure 8J). To determine if contingent administration of a clinically relevant opioid in adulthood is impacted by 3-trimester opioid exposure, saline and NOWS mice underwent 10 days of remifentanil self-administration (0.1 mg/kg/infusions) in 2-h daily sessions. There was no difference between sex or treatment in the number of nose pokes, or the amount of drug infused, during the training, FR1, or FR2 portions of the self-administration test (Figure 8K). Additionally, there was no difference in the progressive ratio breakpoint between NOWS and saline mice (Figure 8L).

### 3-trimester NOWS alters social behavior in adulthood in a sex-dependent manner

**FIGURE 9:**
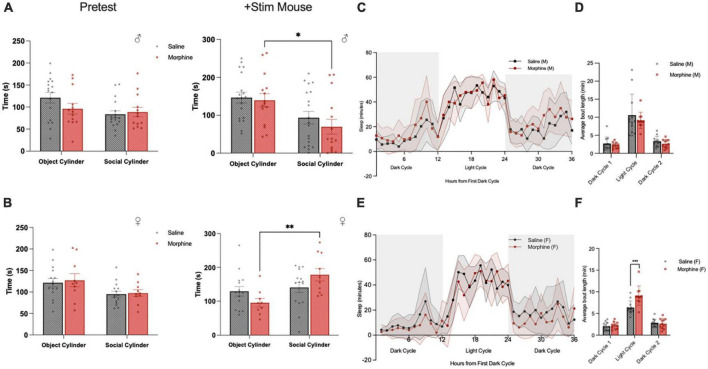
3-Trimester NOWS alters social and sleep behavior in adulthood. **(A)** Time spent interacting with the object-paired and social-paired cylinder in 3-chamber social affiliation test in males during the pretest (empty cylinder phase) and with addition of a stimulus mouse. During the stimulus mouse phase, there was a significant effect of session [F(1, 18) = 13.3, *p < 0.05], but no session x NOWS treatment interaction [F(1, 10) = 0.23, *p* > 0.05]. *Post hoc* analysis revealed morphine males spent significantly less time interacting with the social cylinder than the object cylinder (*p* < 0.05), but there was no significant difference in saline males. **(B)** Time spent interacting with the object-paired and social-paired cylinder in 3-chamber social affiliation test in females during the pretest (empty cylinder phase) and with addition of a stimulus mouse. During the stimulus mouse phase, there was a significant effect of session [F(1, 45) = 8.8, **p < 0.01] and a significant session x NOWS treatment interaction [F(1, 45) = 5.1, *p* < 0.05]. *Post hoc* analysis revealed morphine females spent significantly more time interacting with the social cylinder than the object cylinder (*p* < 0.01), but there was no significant difference in saline males. **(C)** Time spent sleeping, as measured by the COMPASS system, in male mice. There was a significant effect of time [F(35,770) = 38.2, *p* < 0.001], and a significant time x NOWS treatment interaction [F(35,770) = 1.7, *p* < 0.01]. **(D)** Average sleep bout length in each light and dark period in male mice. There was a significant effect of time [F(2,44) = 1.6, *p* < 0.001], but no effect of NOWS treatment. **(E)** Time spent sleeping, as measured by the COMPASS system, in female mice. There was a significant effect of time [F(35,735) = 33.6, *p* < 0.001] and a modest time x NOWS treatment interaction [F(35,735) = 1.4, *p* < 0.1]. **(F)** Average sleep bout length in each light and dark period in female mice. There was a significant effect of time [F(2,52.6) = 139.9, *p* < 0.001], and a significant time x NOWS treatment interaction [F(2,52.6) = 9.3, *p* < 0.001]. *Post hoc* analysis revealed a significant difference in sleep bout length between NOWS and saline mice during the light period [χ2(1, *N* = 23) = 16.54, ***p < 0.001]. All data was generated from 11 total litters (6 saline, 7 NOWS).

While there is no identified association between *in utero* opioid exposure and Autism Spectrum Disorders (ASD) in humans, we had identified an overlap with ASD datasets in our transcriptome analysis detailed above (Figure 4). Therefore, we tested whether 3-trimester opioid exposure and subsequent withdrawal causes specific behaviors associated with neurodevelopmental disorders like ASD (Hol et al., 1996; Niesink et al., 1996). In a 3-chamber social affiliation assay, we found that male NOWS mice displayed a significant preference to interact with the non-social cylinder compared to the social cylinder (Figure 9A). In contrast, female NOWS mice showed a significant preference for the cylinder when the stimulus mouse was present (Figure 9B).

### 3-trimester NOWS alters sleep patterns

To explore other phenotypes associated with ASD, we examined sleep behaviors, which to our knowledge have not previously been assayed in mouse models of *in utero*/post-natal opioid exposure. Sleep was defined as 40 consecutive seconds or more of inactivity as measured by the COMPASS system, which has been shown to correlate with EEG sleep measures in mice (Brown et al., 2016). Male NOWS mice exhibited significantly different sleep-wake patterns than saline mice over a 36-h period that included two dark periods and one light period (Figure 9C). There was no effect in NOWS males in the length of sleep bouts (Figure 9D). However, in female mice, there was no difference in overall sleep patterns between NOWS and saline mice (Figure 9E), but female NOWS mice had significantly longer sleep bouts during the light period compared to saline mice (Figure 9F).

## Discussion

We describe a 3-trimester human equivalent mouse model of *in utero* and post-natal opioid exposure, and subsequent neonatal opioid withdrawal syndrome (NOWS), with unique construct validity. Mice that were exposed to morphine throughout all 3-human equivalent trimesters developed phenotypes that mimic many aspects of human opioid exposure and NOWS, including decreased body weight, delayed early developmental milestones, and a robust physical withdrawal response that included increased vocalizations, somatic disturbances and thermosensitivity. Together, these phenotypes demonstrate that this preclinical model has strong face validity and reproducibility (see Supplementary Figure 1).

A recent review of preclinical NOWS models in which the duration of morphine exposure was limited to gestational exposure did not show changes in body weight or delays in developmental milestones (Byrnes and Vassoler, 2018). However, opioid exposure restricted to the third trimester-equivalent (PND1–14) can produce opioid withdrawal-related phenotypes including thermal hyperalgesia and altered ultrasonic vocalization (USV) (Borrelli et al., 2021). This suggests that while the full, 3-trimester morphine exposure model described herein has more severe developmental effects relative to models with more limited exposure, exposure during the critical third trimester alone may be sufficient to produce withdrawal symptoms. Notably, clinical withdrawal is less prevalent in infants born prematurely, emphasizing the significance of exposure during the third trimester to NOWS (Ruwanpathirana et al., 2015).

Although the variable appearance of NOWS in infants exposed to opioids *in utero* is incontrovertible, and preclinical models demonstrating spontaneous withdrawal are accumulating, the presence or absence and nature of long-term effects remain controversial (Conradt et al., 2019). Recent studies show perinatal opioid exposure impacts affective behaviors in adolescence (Alipio et al., 2021, 2022), but long term effects in adulthood are not observed (Minakova et al., 2022). Challenges in identifying effects of prenatal opioids in a clinical setting include the wide variability in choice of opioid, the presence of polypharmacy in a majority of opioid-addicted mothers, and the varying socioeconomic circumstances of the children (Weller et al., 2021). Importantly, behavioral deficits are observed in children exposed to opioids *in utero* but raised by an adoptive parent (Ornoy et al., 2001), demonstrating that early opioid-induced alterations in CNS function may supersede parental environment as a critical factor. Thus, preclinical models are useful in the delineation of prenatal “opioid only effects.” However, even rodent models are plagued by wide variability, including, but not restricted to, choice of opioids, mode, level, and duration of exposure, care of litters, and choice of behavior assays. For example, there are animal models of prenatal opioid exposure demonstrating long-term anxiogenic, depressive and reward-altering effects (Ahmadalipour et al., 2015), while others have reported the opposite or no effect (Craig and Bajic, 2015). One review noted that most observations of long-term effects are not consistent, and when they were detected, were often relatively small and only found in one sex (Fodor et al., 2014). It is also possible that the rodent assays being used by investigators, ourselves included, do not detect what are perhaps becoming the most recognized deficits in children exposed *in utero* to opioids, namely, hyperactivity, attention deficits, and overall “behavior problems” (Boggess and Risher, 2022).

Our model showed no significant differences in affective or opioid-related behaviors tested in adulthood, despite the high levels of morphine exposure during development and the robust withdrawal phenotype. However, we did observe alterations in social affiliative behavior. While males NOWS mice showed a significant decrease in social preference, female NOWS mice displayed a significant increase in social preference, indicating a potentially adaptive response after the 3-trimester exposure and subsequent withdrawal. Of note, saline treated mice in this assay did not exhibit the expected social affiliation in this assay (Figure 9A). However, when naïve mice were tested, trends for the expected social affiliative behavior were observed (Supplementary Figure 8). As our model involves twice-daily injections through PND14, the lack of response in saline treated mice may be an effect of early-life injection stress. Regardless, early life exposure with morphine clearly alters social behavior in both males and females.

Female NOWS mice had significantly longer sleep bouts than saline female mice, and fragmented sleep is associated with poor health and inflammation (Trammell et al., 2014), suggesting an adaptive response of sleep patterns. One recent study of early-life maternal separation and handling stress demonstrated that early-life stress increased social preference in male and female mice (Bondar et al., 2018); however, overall there are very few preclinical or clinical studies investigating positive adaptations after prenatal opioid exposure, or other early-life stressors.

In addition to the long-lasting alterations in social behavior, we report the first comprehensive RNA sequencing analysis of the *in vivo* transcriptomic effects in the forebrain of *in utero* opioid exposure in mice. These datasets are highly novel in that they directly compare three commonly used exposure protocols, i.e., *in utero* only (PND1), post-natal only (PND 14), and a combination of the two (3-Tri). Recently, there have been attempts to use translationally relevant models of NOWS both in terms of drug(s) of choice and duration of exposure, and although human exposure generally encompasses all 3 trimesters, there are increased attempts to detoxify pregnant patients in place of opioid agonist therapy (Macfie et al., 2020). Thus, knowing the extent of gene expression changes at various times throughout pregnancy is important. Despite the minimal amplitude of gene alterations, these data were in line with the few other RNA seq data derived from animals with *in utero* or post-natal opioid exposure (Odegaard et al., 2020; Borrelli et al., 2021). Although we were concerned that using whole hemisphere RNA would obscure regional DEGs, it is notable that despite targeted sequencing, either from brainstem or nucleus accumbens, our data conforms to many of the same identified pathways as those identified in previously published studies.

We compared our pathway gene ontology, and differential expression (DE) analyses to curated datasets from the literature. These data were mostly derived from adult animals, either analyzing gene expression following chronic opioid exposure and/or withdrawal (Zhang et al., 2017; Yuferov et al., 2018; Zhang Y. et al., 2018; Odegaard et al., 2020; Borrelli et al., 2021; Jiang et al., 2021; Liu et al., 2021; Mendez et al., 2021; Radhakrishna et al., 2021; Seney et al., 2021; Townsend et al., 2021; Coffey et al., 2022; Corradin et al., 2022; Fox et al., 2022; Toorie et al., 2022). The following cutoffs were used to filter datasets in the literature: IPA pathways [−log(*p*-value) > 1.3], GO terms (*p*-value < 0.05), and DEGs (nominal and/or adjusted *p*-value < 0.05). For the IPA and GO analysis, we searched available datasets for canonical pathways and GO terms that contained keywords related to our study and met our cutoff criteria.

Comparing canonical pathways, the CREB Signaling pathway, Opioid Signaling pathway, Synaptogenesis, Ephrin Receptor Signaling, and Semaphorin Neuronal Repulsive Signaling were enriched among differentially expressed gene in our data and in published studies pertaining to opioid exposure and withdrawal. When analyzing upstream regulators, CREB1 stands out in the PND1 and PND14 groups, but is less important in the 3-trimester exposure paradigm. CREB is a well characterized transcriptional regulator associated with opioid exposure and behavior in adulthood (Blendy and Maldonado, 1998; Walters et al., 2005; Lardner et al., 2021), and thus its prevalence following *in utero* or PND14 exposure suggests important roles in developmental opioid exposure as well. Interestingly, TP53 was identified in all six of our datasets, and was highlighted in a study from human adult brains with a focus on angiogenesis and cytokine signaling (Mendez et al., 2021). Although cytokine signaling was not directly obvious in our datasets, phagosome formation was a prominent IPA pathway, implicating a microglial response, as does TGFB1 as an upstream regulator. The identification of levodopa as an upstream regulator is of course consistent with the long recognized key role of the neurotransmitter dopamine in opioid effects (Koob, 1992).

Similar to IPA canonical terms, there was overlap of multiple GO terms identified in our data with those in the literature. These included the GO biological process “Neuron-neuron Synaptic Transmission” which appeared in more than one publication, highlighting the role of synaptic plasticity in response to opioids at all ages. GO terms relating to mitochondrial functioning, regulation of membrane potential, immune response, angiogenesis, and extracellular matrix organization also overlapped with multiple reports. Finally, we searched for DEGs that appeared more than once in the literature and found using the adjusted *p*-value as a cutoff that only 6 genes (*Fnbp1l, Hspa5*, *Mmp17*, *Scn2b, Serpinh1*, *Slc6a6*) overlapped with DEGs from two publications. Regional, bulk cell-type, and single cell sequencing are likely to highlight more specific pathways in the future, for example, in the dopamine and microglial systems. We conclude that *in utero* or early post-natal exposure to specific opioids has the capacity to impact neuronal circuity (Salzwedel et al., 2020; Radhakrishnan et al., 2022; Vishnubhotla et al., 2022) with long-lasting effects in select behaviors that outlast the alterations to gene activation. The comparisons of perinatal and adult datasets highlight the large number of diverse genes that contribute to various pathways, so that phenotypic responses may overlap considerably more than transcriptional responses. Notably, an unbiased transcriptomics approach led us to assay specific behaviors which turned out to be impacted by morphine exposure, i.e., social preference and sleep patterns. The overlap with ASD genetic and transcriptome studies are excellent clues warranting further investigation into specific genes, pathways, and mechanisms which might be impacting these behaviors in NOWS. In addition to the relevance of GABA pathways discussed above, perturbations in mitochondrial function and oxidative phosphorylation are increasingly recognized as sources of neurodevelopmental abnormalities as they play a role in multiple processes, including neurogenesis, cell migration, and axonal development (Lin-Hendel et al., 2016) and interneuron development (Khacho et al., 2019). A large proportion of the interneurons are GABAergic, thereby linking some of the pathways. RNA sequencing was performed on equal numbers of male and female samples, so the differences between sexes in the RNA sequencing results are intriguing, as the sex-dependent vulnerability to neonatal opioid withdrawal syndrome requiring therapy and/or long-term effects of *in utero* opioid exposure remain as open questions (Byrnes and Vassoler, 2018; Weller et al., 2021) despite the fact that pre-clinical models almost all show sex differences.

We used morphine, a short-acting full opioid agonist, to model continuous opioid exposure. The long-term effects identified here may differ from those caused by the partial agonist buprenorphine, or the long-lasting agonist methadone, which are used to treat opioid use disorder during pregnancy. In fact, specific differences between these drugs on the impact of *in utero*/perinatal exposure on myelination have already been documented (Velasco et al., 2021). Although there is a move toward non-pharmacologic interventions, opioid maintenance and taper are still widely used for acute, symptomatic NOWS in humans. It is unclear whether the long-term effects identified here are caused by opioid exposure itself, or the negative early-life experience of withdrawal. Future studies investigating the effect of treating withdrawal symptoms in this paradigm will be important for answering these mechanistic questions and understanding how to better prevent potential long-term consequences of NOWS.

We found here that NOWS affects neurodevelopment in a way that is similar to many other early-life exposure models, including early-life stress, maternal immune activation, and maternal high fat diet, leading to changes in social and sleep behavior. Notably, the severe NOWS phenotype in our model did not cause any changes in adult drug seeking or affective behaviors. In fact, we would argue that the gene expression changes, and long-term behavior consequences were milder than anticipated. These data imply that *in utero* exposure to opioids alone may not account for many of the abnormalities noted in clinical practice, and additional research is necessary to study the effects on the fetus and the molecular mechanisms associated with polypharmacy in pregnant patients.

## Data availability statement

The datasets presented in this study can be found in online repositories. The names of the repository/repositories and accession number(s) can be found in the article/Supplementary material.

## Ethics statement

The animal study was reviewed and approved by the University of Pennsylvania Animal Care and Use Committee and were in compliance with the National Institutes of Health Guide for the Care and Use of Laboratory Animals.

## Author contributions

AD, SR, MEE, and JAB conceived and designed the experiments. AD, SR, CL, JM, and JF performed the experiments and analyzed the data. JC-M, CN, ME, and AR performed -omics analysis. ML and JH performed morphine measurements. JKB contributed statistical analysis. AD, MEE, and JAB wrote the manuscript. SR and JF edited and provided critical feedback on the manuscript. All authors contributed to the article and approved the submitted version.

## Footnotes

1
https://github.com/shenlab-sinai/NGS-Data-Charmer

2
https://www.bioinformatics.babraham.ac.uk/projects/trim_galore/

3
https://broadinstitute.github.io/picard/

4
https://digitalinsights.qiagen.com/IPA
