# Supplementary material for: Molecular and long-term behavioral consequences of neonatal opioid exposure and withdrawal in mice
Source: Front Behav Neurosci. 2023 Jun 21;17:1202099. doi: 10.3389/fnbeh.2023.1202099 (PMC10324024; doi:10.3389/fnbeh.2023.1202099)
Supplement: Supplementary file 1 [file Data_Sheet_1.PDF]

Developmental Milestones | **Withdrawal**

Figure 1 Cohort 1	Sal	Mor
Females	16	18
Males	9	24

	Body Weight	Forelimb Grasp	Eye Opening	Extinguish Pivoting	Surface Righting
Females	ns	***	ns	*	ns
Males	*** (time x treatment)	ns	*	****	**

2-way ANOVA (Body Weight); t-test (Forelimb Grasp to Surface Righting)

Figure 1 Cohort 1	Sal	Mor
Mixed Sexes	25	42

Somatic Withdrawal	Hotplate Latency	USV Bins
8 hours: ns<br>24 hours: **<br>48 hours: ns	8 hours: *<br>24 hours: **<br>48 hours: ns	8 hours: **<br>24 hours: **<br>48 hours: **
Overall: * (time x treatment)	Overall: ***	Overall: *

Linear Mixed-Effects Model

Figure 8 Cohort 2	Sal	Mor
Females	14	6
Males	5	11

	Body Weight	Forelimb Grasp	Eye Opening	Extinguish Pivoting	Surface Righting
Females	***	ns	ns	ns	*
Males	****	**	*	*	*

Somatic Withdrawal	Hotplate Latency	USV Bins
ns	****	ns
ns	**	*

Figure 8 Cohort 3	Sal	Mor
Females	4	17
Males	3	15

	Body Weight	Forelimb Grasp	Eye Opening	Extinguish Pivoting	Surface Righting
Females	****	**	ns	ns	ns
Males	****	*	ns	*	*

Somatic Withdrawal	Hotplate Latency	USV Bins
ns	ns	*
ns	**	**

Figure 9 Cohort 4	Sal	Mor
Females	16	11
Males	19	15

	Body Weight	Forelimb Grasp	Eye Opening	Extinguish Pivoting	Surface Righting
Females	**	N/A	N/A	N/A	N/A
Males	****	N/A	N/A	N/A	N/A

2-way ANOVA (Body Weight); t-test (Forelimb Grasp to Surface Righting)

Somatic Withdrawal	Hotplate Latency	USV Bins
N/A	**	*
N/A	*	****

t-test

Supplemental Figure 1. Characteristics and summary of NOWS phenotypes in all cohorts. A total of 4 different cohorts were tested used to generate data for this study. Each figure and the corresponding cohort numbers are listed on the left. Effects of full 3-trimester opioid exposure on body weight, developmental milestones and withdrawal for both sexes in all cohorts are shown here. Sexes were combined for withdrawal in Figure 1, but otherwise tested separately. *$p<0.05$, **$p<0.01$, ***$p<0.001$, ****$p<0.0001$.

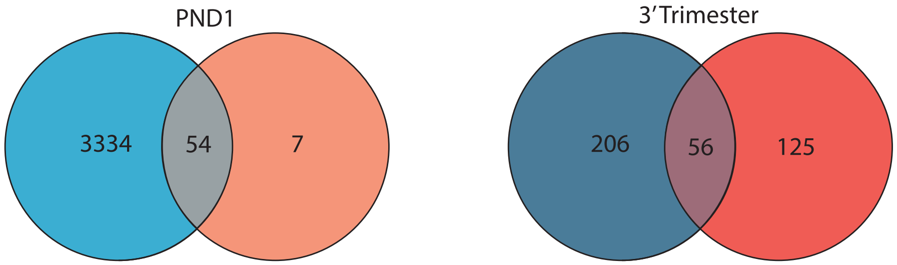

Supplemental Figure 2 Venn diagrams Low levels of DEG (Adj p value <0.05) across treatment group (labeled) and across sex (blue = male; orange = female)

Supplemental Fig 3:
Top 3 Gene Ontology Biological Processes Up-regulated processes (red) and and Down-regulated processed (blue)
in females from all three exposure protocols, cutoff pAdj<0.05 for DEGs, showing adjPvalue on X axes, cutoff = 1.2.

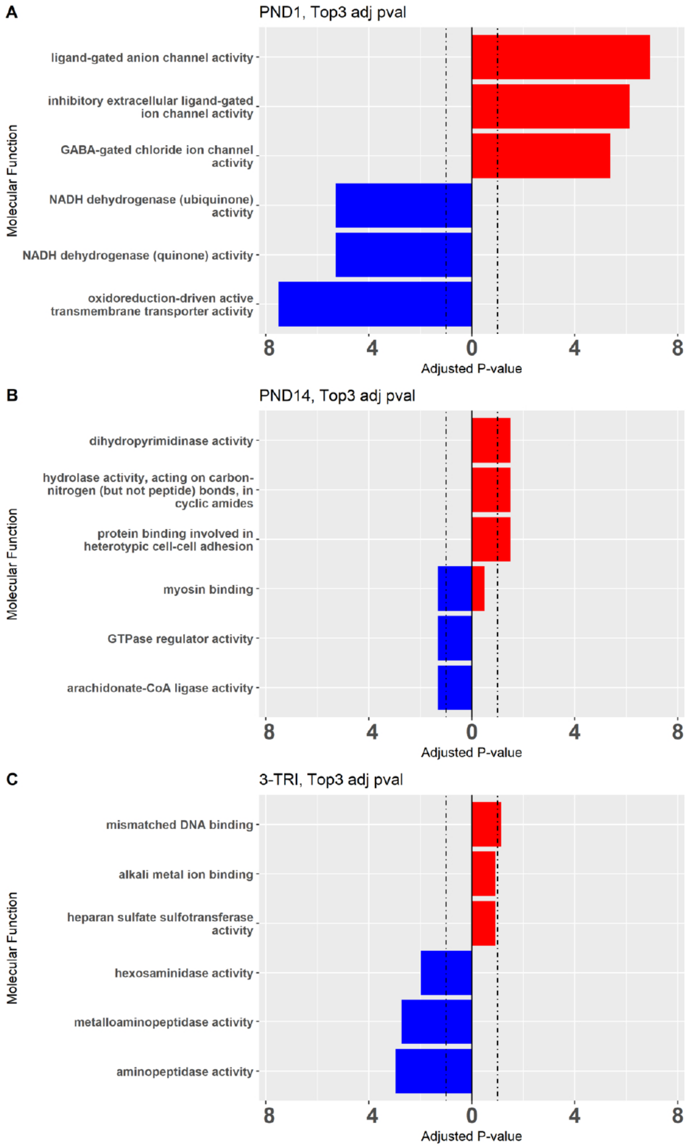

GO Molecular

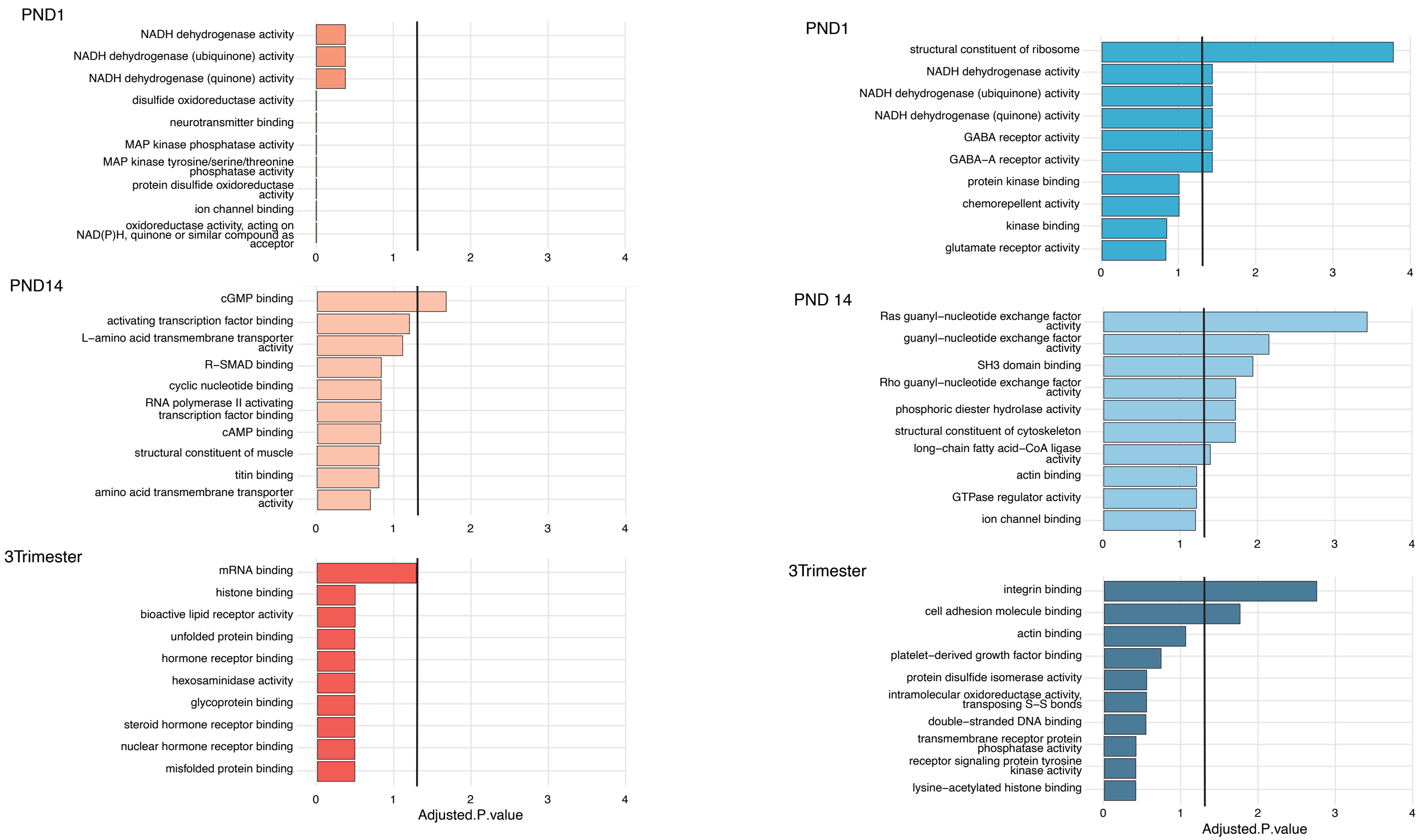

Supplemental Figure 4. Gene Ontology (A) Top 10 Gene Ontology Molecular Processes for males (blue) and female (orange) DEG datasets, showing adjPvalue on X axes, cutoff = 1.2.

GO Cellular

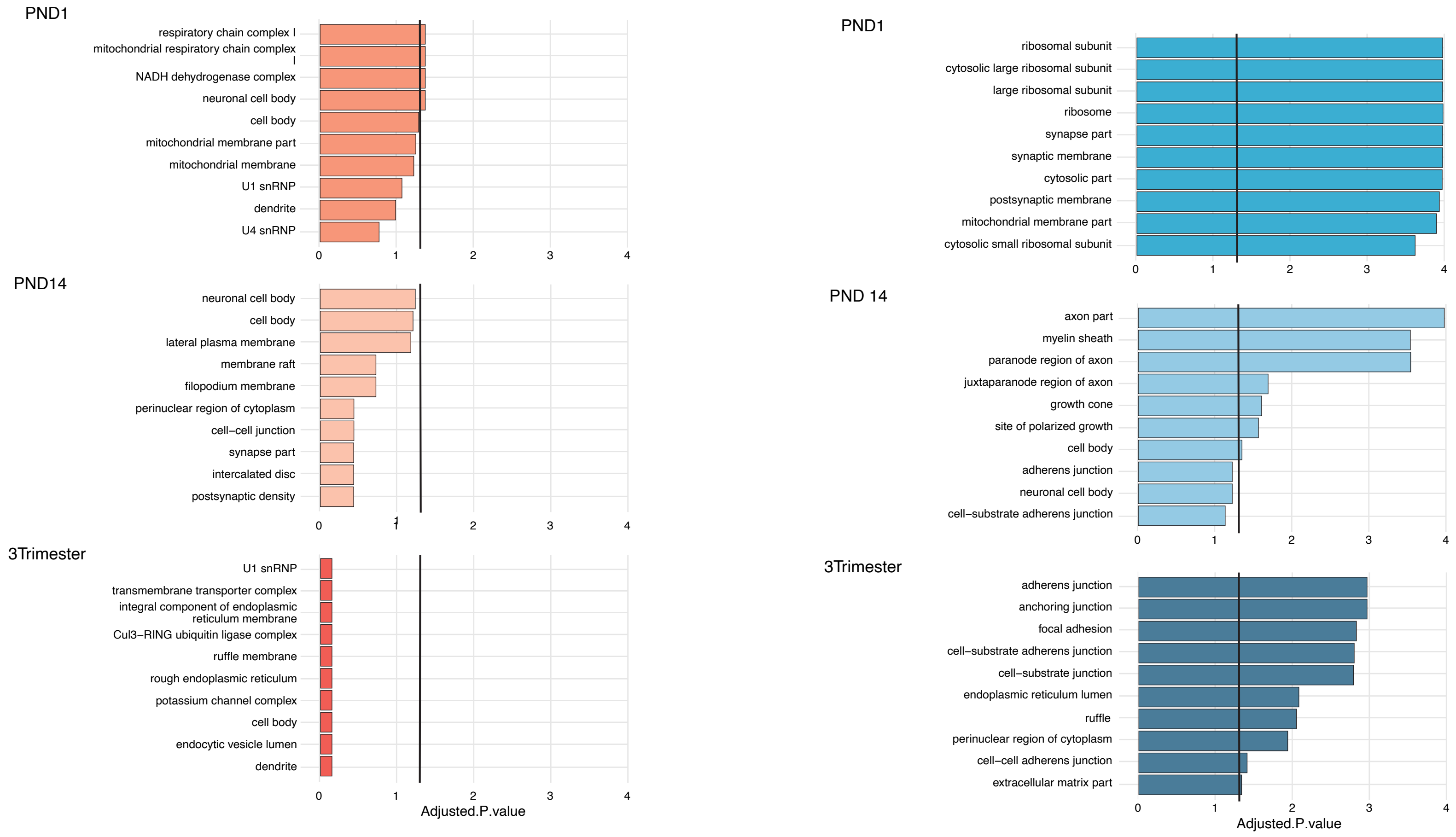

Supplemental Figure 5. Gene Ontology(**B**) Top 10 Gene Ontology Cellular Components for males (blue) and female (orange) DEG datasets from all three treatment protocols, showing adjPvalue on X axes, cutoff = 1.2.

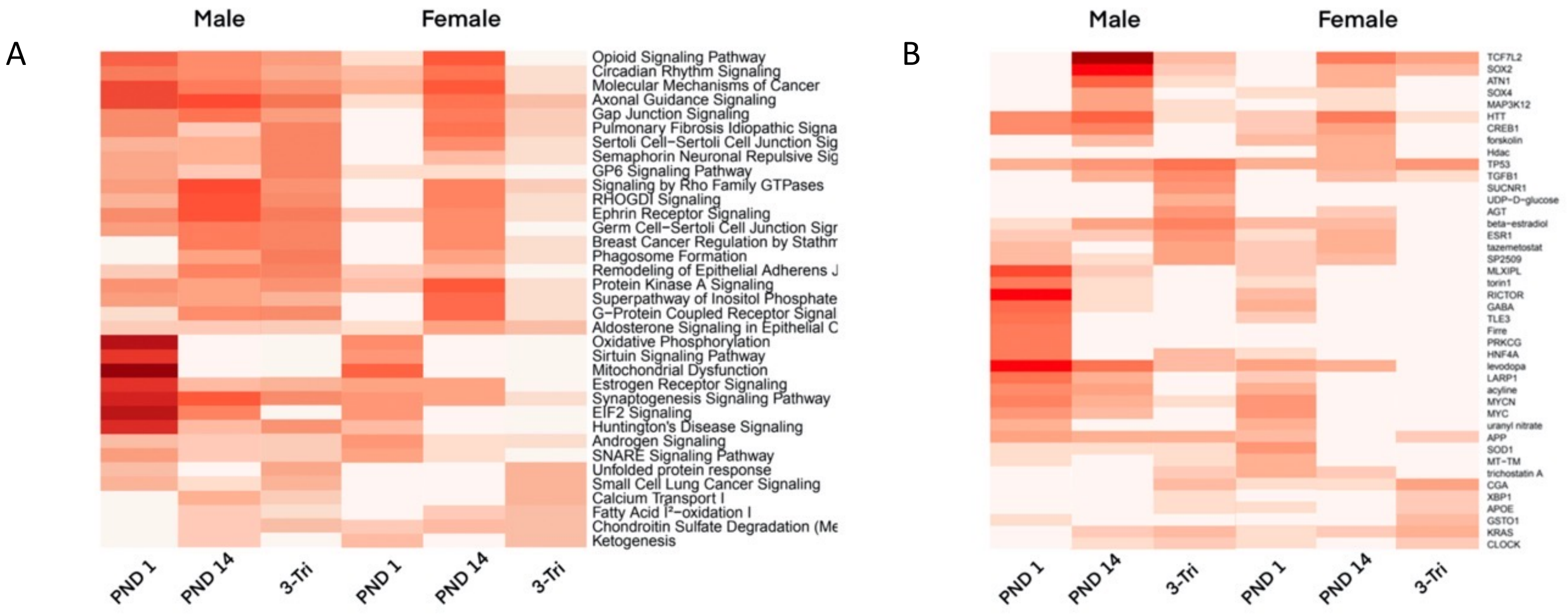

Supplemental Figure 6 Pathway Analysis (A) Top IPA canonical pathways across exposure protocols and sex. Heatmap of adjusted P-values, with rows representing Canonical pathways, and the exposure protocol and sex-segregated models shown as columns. The negative log10 of the adjusted P-values is shown in shades of red, with darker reds representing a smaller p-value. **(B)** Top IPA Upstream Regulators across exposure protocols and sex. Heatmap of adjusted P-values, with rows representing Canonical pathways, and the exposure protocol and sex-segregated models shown as columns. The negative log10 of the adjusted P-values is shown in shades of red, with darker reds representing a smaller p-value.

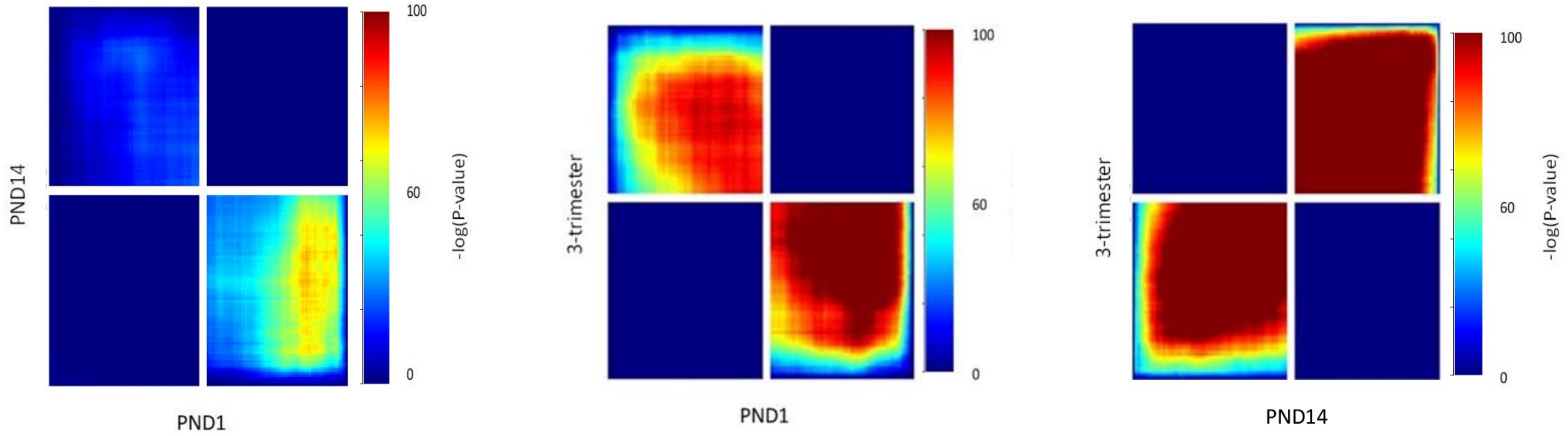

Figure 7 : RRHO analysis of male datasets showing pair-wise comparisons of the three morphine exposure protocols RRHO plot shows lack of concordance between PND1 and PND14, a moderate degree of discordance between PND1 and 3-trimester, and a high degree of concordance between PND14 and 3-trimester.

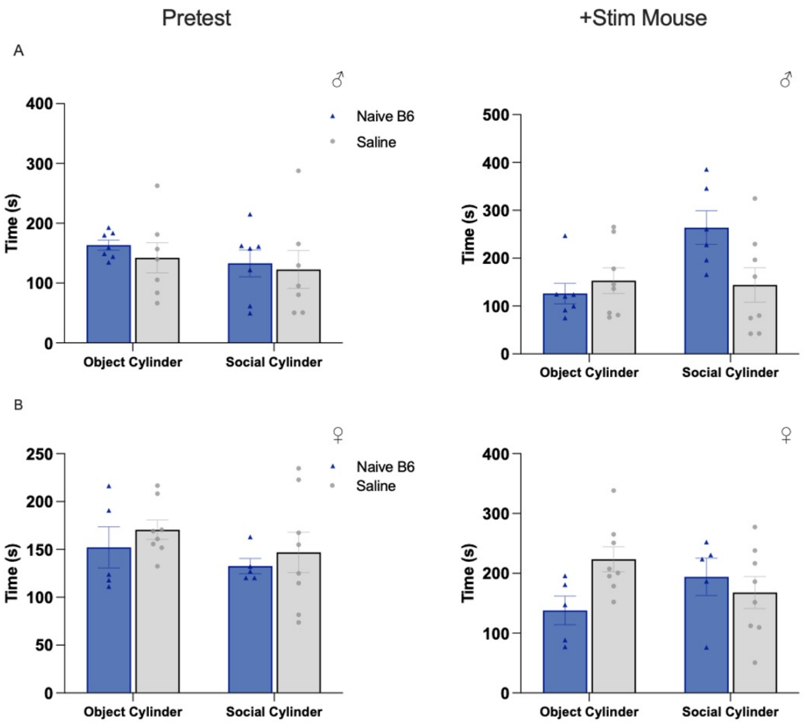

Supplemental Figure 8. Effect of NOWS paradigm injections on social behaviors. Saline mice from an additional cohort (not shown in Supp Figure 1) were tested alongside naïve C57B6 mice from the same colony for social behaviors. Time spent interacting with the object-paired and social-paired cylinder in 3-chamber social affiliation test during the pretest (empty cylinder phase) and with addition of a stimulus mouse. in a 3-chamber social affiliation test in males (**A**) and females (**B**). In both sexes there was a non-significant trend for the naïve mice to spend more time interacting with the social cylinder.
